# Accuracy of Endoscopic Ultrasonography for Determining the Depth of Invasion in Early Gastric Cancer

**DOI:** 10.5152/tjg.2022.21847

**Published:** 2022-09-01

**Authors:** Seung-Jun Kim, Chul-Hyun Lim, Bo-In Lee

**Affiliations:** 1 Department of Gastroenterology, H Plus Yangji Hospital, Seoul, Republic of Korea; 2 Division of Gastroenterology, Department of Internal Medicine, Seoul St. Mary’s Hospital, The Catholic University of Korea Faculty of Medicine, Seoul, Republic of Korea; 3 Division of Gastroenterology, Department of Internal Medicine, Eunpyeong St. Mary’s Hospital, The Catholic University of Korea Faculty of Medicine, Seoul, Republic of Korea

**Keywords:** Endoscopic mucosal resection, endosonography, gastrectomy, stomach neoplasms

## Abstract

**Background:**

Accurate staging for depth of invasion (T stage) of early gastric cancer is critical for determining the treatment modality. Endoscopic ultrasonography is a reliable method for assessing the T stage. However, its diagnostic accuracy varies. The aim of this study is to investigate clinicopathologic factors affecting the diagnostic accuracy of endoscopic ultrasonography in early gastric cancer.

**Methods:**

Patients with early gastric cancer who had undergone endoscopic resection or gastrectomy were included. The diagnostic accuracy of endoscopic ultrasonography was evaluated by comparing the T stage by endoscopic ultrasonography with histopathology of the resected specimen. Subgroup analysis was performed according to the endoscopic resection criteria.

**Results:**

A total of 223 early gastric cancer lesions were included. The overall accuracy of endoscopic ultrasonography for T staging was 66.4%. The diagnostic accuracy for lesions ≤2 cm was significantly higher than for those of 2-3 cm (odds ratio 3.59) or those >3 cm (odds ratio 5.47). The diagnostic accuracy was significantly decreased in lesions with ulceration (odds ratio 2.62) or non-flat morphology (odds ratio 2.94). The accuracy of endoscopic ultrasonography for lesions corresponding to the absolute endoscopic resection criteria was significantly higher than for those corresponding to the expanded criteria (97.3% vs 71.9%, *P =* .002). Of the tumors that were overestimated by endoscopic ultrasonography treated with gastrectomy, 93.3% corresponded to the expanded criteria.

**Conclusion:**

Endoscopic ultrasonography had poor accuracy in early gastric cancer lesions larger than 2 cm, those with ulceration, and those with non-flat morphology, that is, lesions corresponding to the expanded criteria were more frequently overstaged by endoscopic ultrasonography. Such early gastric cancers should be carefully considered when staging by endoscopic ultrasonography before gastrectomy.

**Main Points**

- In early gastric cancer (EGC), tumor size larger than 2 cm, ulceration, and non-flat morphology decreased the accuracy of endoscopic ultrasonography (EUS).
- Expanded criteria lesions were more frequently overstaged by EUS.
- Lesions corresponding to the expanded criteria should be more carefully diagnosed by EUS to avoid overtreatment with gastrectomy.

## Introduction

Early gastric cancer (EGC) is defined as gastric cancer in which invasion is limited to either the mucosal or submucosal layers, regardless of the presence of lymph node involvement.^1,2^ The Korean National Cancer Screening Program increased the detection rate of EGC to 70% by providing individuals over 40 years of age with upper gastrointestinal endoscopy every 2 years.^3,4^ As a result, determining the optimal treatment modality of EGC has become crucial.

With the development of endoscopic interventions, endoscopic treatment, such as endoscopic mucosal resection and endoscopic submucosal dissection, is widely performed in clinical practice for EGC, as it is less invasive, more convenient, and more efficient in the long term.^5^ The criteria for endoscopic treatment of EGC have been extended to well-differentiated, non-ulcerative tumors >20 mm, ulcerative tumors ≤30 mm, and SM1 (penetration into the submucosal layer <500 µm from the muscularis mucosa) tumors ≤30 mm.^6,7^ Additionally, recent guidelines recommend endoscopic treatment for undifferentiated, non-ulcerative tumors ≤20 mm in diameter, which have a low incidence of extragastric metastasis during follow-up.^8,9^ Accordingly, the prediction of the depth of invasion (T stage) is important.

Endoscopic ultrasonography (EUS) is the first-choice diagnostic modality for assessing the T stage in EGC because of its high diagnostic rate. It is superior to multidetector computed tomography (MDCT) or magnetic resonance imaging, especially in distinguishing mucosal lesions from submucosal lesions.^10,11^ Endoscopic ultrasonography is recommended as a critical diagnostic tool for superficial gastrointestinal neoplasia with suspicious features to detect submucosal invasion.^8,9^ However, findings on the accuracy of EUS in diagnosing the T stage are conflicting. The accuracy of EUS varies from 65.0% to 92.1%^11^ and is significantly affected by endoscopic findings such as ulceration, lesion location, disease stage, tumor size, and research design.^12-17^

The accuracy of EUS is important in that it allows clinicians to determine whether endoscopic resection is the correct choice of treatment. Investigating clinicopathologic factors thus contributes to the high diagnostic efficacy of EUS. The aim of this study is to evaluate the accuracy of EUS in predicting the depth of invasion in EGC and to analyze clinicopathologic factors affecting the diagnostic accuracy of EUS in EGC.

## MATERIALS AND METHODS

A retrospective trial was performed at a single academic hospital from March 2016 to September 2019. The study protocol was approved by the Institutional Review Board and conformed to the 1964 Helsinki declaration and its later amendments or comparable ethical standards.

### Patients and Study Design

All patients with proven histopathologic EGC, defined as invasive gastric that invades no more deeply than the submucosa, were enrolled and administered curative treatment by endoscopic resection or gastrectomy. If endoscopic resection was confirmed as incomplete, additional gastrectomy was performed. Before treatment, patients underwent conventional esophagogastroduodenoscopy (EGD), EUS, and MDCT for T staging. Patients were excluded if the lesion was accompanied by histopathologic confirmation of invasion of the muscularis propria. The medical records of patients, including information such as age, gender, and body mass index, were reviewed, and the final histopathologic staging was compared to the pretreatment staging by EUS. Subgroup analysis was also performed according to the endoscopic resection criteria.

### Procedure and Endoscopic Ultrasonography staging

All patients underwent conventional EGD followed by EUS. Endoscopic ultrasonography examination was performed by experienced endoscopists who are qualified by the Korean Society of Gastrointestinal Endoscopy with more than 2 years of experience using the radial and/or mini-ultrasonic probe incorporating a scanning system with a frequency of 5-20 MHz (GF-UM2000^®^, UM-DP12-25R^®^ or UM-DP20-25R^®^; Olympus Co.: Tokyo, Japan). Patients were premedicated with local pharyngeal lidocaine anesthesia (Veracaine Spray^®^, Firson Co.: Chungcheongnam-do, Republic of Korea), pethidine (25-50 mg intravenous; Pethidine HCl^®^, Myungmoon Pharm Co.: Seoul, Republic of Korea), and midazolam (2-6 mg intravenous; Midazolam^®^, Bukwang Pharm Co.: Seoul, Republic of Korea). Endoscopic ultrasonography images were obtained by instilling deaerated water in the gastric lumen or by a water-filled balloon on the instrument’s tip.

**Figure 1.**
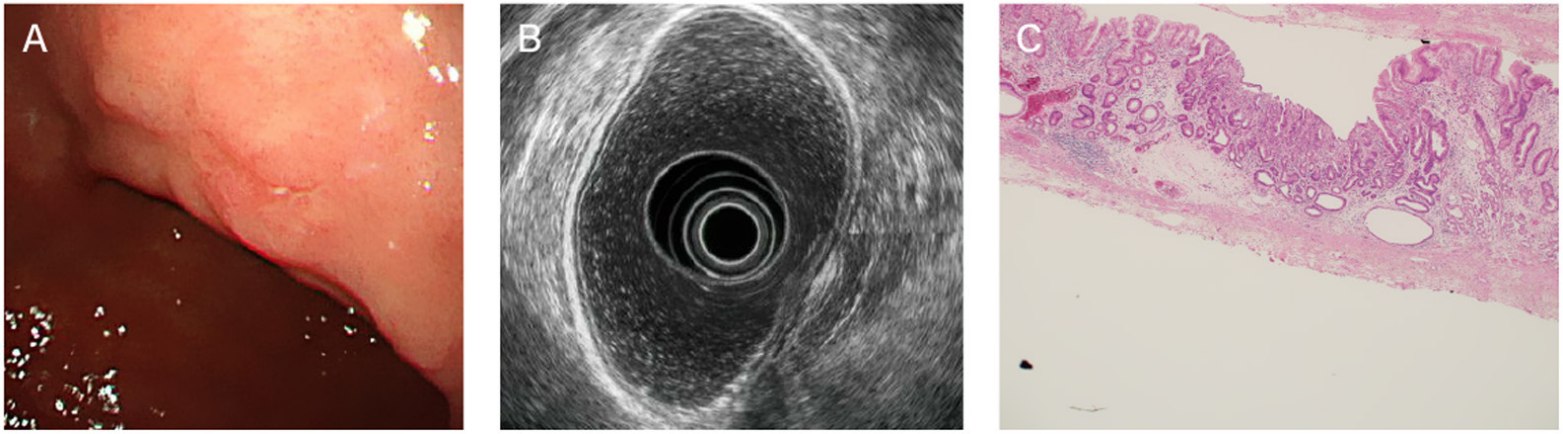
Endoscopic and pathologic figure of mucosal cancer. (A) The lesion was observed with white-light endoscopy; (B) EUS reveals a hypoechoic tumor regarded as the mucosal layer; (C) the histological examination after endoscopic resection demonstrated that the tumor was limited to the mucosal layer (T1a). EUS, endoscopic ultrasonography.

**Figure 2.**
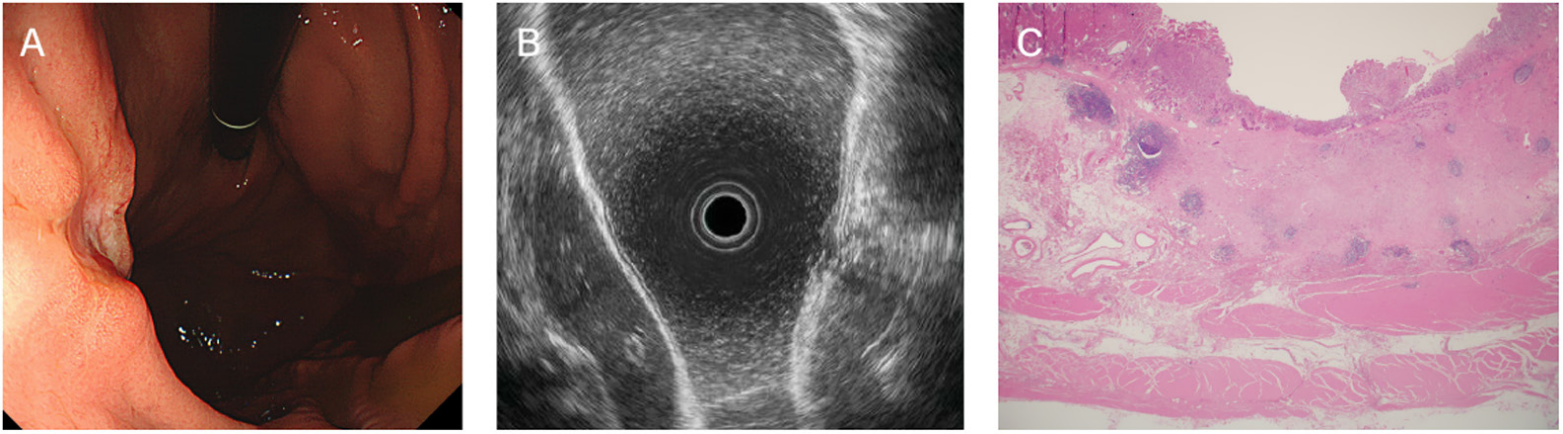
Endoscopic and pathologic figure of submucosal cancer. (A) The lesion was observed with white-light endoscopy; (B), EUS reveals a hypoechoic tumor disrupting the submucosal layer; (C) the histological examination after endoscopic resection demonstrated that the tumor invaded the submucosal layer (T1b). EUS, endoscopic ultrasonography.

On EUS, the combination of the first hyperechoic and second hypoechoic layers corresponded to the mucosa, the third hyperechoic layer to the submucosa, the fourth hypoechoic layer to the muscularis propria, and the fifth hyperechoic layer to the serosa.^18^ A lesion with irregularity, thickness, smooth tapering, or abrupt interruption of the submucosal layer was considered tumor invasion.^19^ Assessment of the depth of invasion by EUS was classified according to the TNM classification. T1a was defined as the tumor invading the first and second mucosal layers, T1b as the tumor invading the third submucosal layer, T2 as the tumor invading the muscularis propria in the fourth layer, T3 as the tumor invading the subserosa without interruption of the serosa, T4a as the tumor invading the serosa in the fifth layer, and T4b as the tumor invading adjacent organs. The macroscopic types were classified using the Japanese Classification of Gastric Carcinoma as type 0-I (protruded type), type 0-IIa (superficial and elevated), type 0-IIb (flat), type 0-IIc (superficial and depression), and type 0-III (excavated).^20^ The location of the EGC lesion was divided into upper, middle, and lower sections based on the Japanese Classification of Gastric Carcinoma^20^ (Figures 1 and 2).

### Histopathologic Evaluation

Histopathologic examination of the resected specimen was conducted in parallel 2-mm-thick sections stained with hematoxylin and eosin. The resected specimens were reviewed by an experienced pathologist who is a gastrointestinal specialist. With regard to the degree of differentiation, well- or moderately differentiated tubular adenocarcinoma and papillary adenocarcinoma were classified as differentiated, and poorly differentiated tubular adenocarcinoma and signet-ring cell carcinoma were classified as undifferentiated. T1b stage was stratified into 2 layers: SM1 and SM2 (penetration over 500 µm).

### Statistical Analysis

Chi-square test or Fisher’s exact test were used to test for associations among categorical variables. A multivariate logistic regression analysis was performed to identify the variables that were considered to have an influence on the accuracy of the depth of invasion by EUS. All data analyses were performed with Statistical Package for the Social Science for Windows version 24 (IBM Corp.; Armonk, NY, USA).

## Results

### Baseline Characteristics

A total of 223 EGCs from 211 patients were analyzed. The mean age of the patients was 62.8 years (±11.2 years), and there were 148 men (66.4%). The mean tumor diameter was 2.6 cm (±1.7 cm), and ulceration was found in 82 tumors (36.8%). In terms of the histopathological diagnosis, 123 tumors were differentiated (55.2%) and 100 were undifferentiated (44.8%). Endoscopic resection was initially performed in 65 (29.1%) patients, of whom 12 with submucosal invasion underwent additional treatment. The patient and lesion characteristics are summarized in Table 1.

### Clinical Factors Affecting the Accuracy of Endoscopic Ultrasonography

The overall accuracy of EUS for T stage was 66.4% (148/223). The accuracies of EUS for T1a and T1b were 70.1% (94/134) and 60.6% (54/89), respectively. The accuracy of EUS was significantly associated with ulceration (odds ratio [OR]: 2.62; 95% CI: 1.38-4.97, *P* = .006), tumor size (2-3 cm: OR: 3.59; 95% CI: 1.65-7.79, *P* = .001; >3 cm: OR: 5.47; 95% CI: 2.55-11.74, *P* < .001), and non-flat morphology (OR: 2.94; 95% CI: 1.11-7.74, *P* = .029). However, tumor location and histopathologic type did not significantly affect the accuracy of EUS. Other factors such as body mass index, age, and gender were also not correlated with the accuracy of EUS (Tables 2 and 3).

**Clinical Factors Affecting the Overestimation and Underestimation of T Stage by **Endoscopic Ultrasonography

Tumors with a larger size, ulceration, and non-flat morphology were overestimated by EUS. In the multivariate logistic regression analysis, tumors 2-3 cm (OR: 4.48; 95% CI: 1.84-10.91, *P* = .001) and those >3 cm (OR: 6.23; 95% CI: 2.58-15.03, *P* < .001) had a significantly higher probability of overestimation than those ≤2 cm. Ulcerative tumors were also more likely to be overestimated (OR: 4.37; 95% CI: 2.13-8.94, *P* < .001). Conversely, non-flat morphology did not affect the overestimation. The underestimation of the T stage was not correlated with tumor size, location, ulceration, macroscopic type, or histopathologic type (Tables 4 and 5). Fifty-nine T1a tumors were overestimated as T1b or T2b. Gastrectomy was used to treat 15 (25.4%) of these tumors, and in 14 (93.3%), the final histopathological result corresponded to the expanded criteria.

### Accuracy of Endoscopic Ultrasonography According to the Criteria for Endoscopic Resection

**Figure 3.**
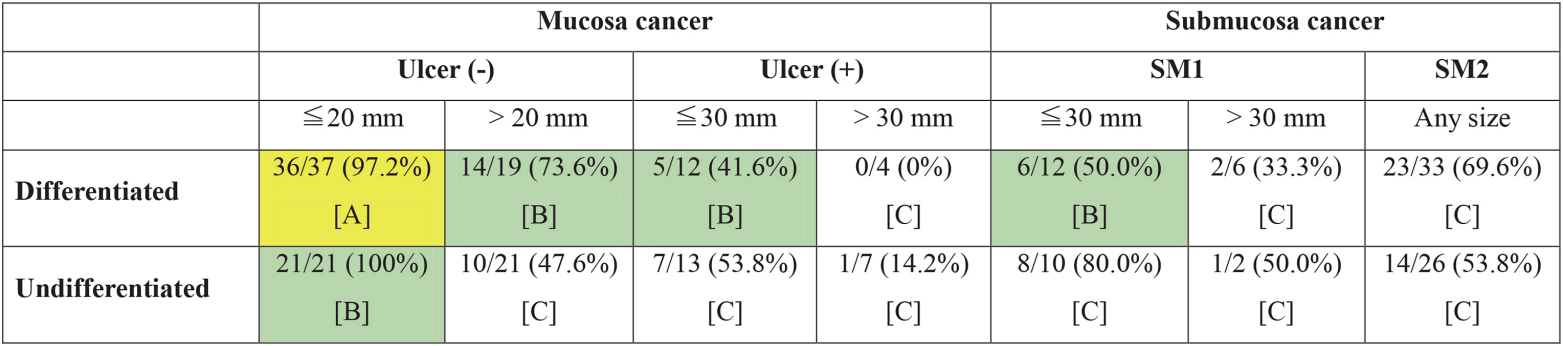
Accuracy of EUS according to the criteria of endoscopic resection. (A) Guideline criteria for EMR/ESD; (B) expanded criteria for EMR/ESD; (C) surgery. EUS, endoscopic ultrasonography; SM1, penetration into the submucosal layer <500 µm from the muscularis mucosa; SM2, penetration over 500 µm; EMR, endoscopic mucosal resection; ESD, endoscopic submucosal dissection.

The accuracy of EUS was reevaluated according to the absolute and expanded endoscopic resection criteria. The accuracy of EUS for lesions corresponding to the absolute criteria (97.3%; 36/37) was significantly higher than that for those corresponding to the expanded criteria (71.9%; 46/64, *P* = .002) (Figure 3).

## Discussion

This study investigated the clinical factors that influence the accuracy of EUS. The results showed that tumor size larger than 2 cm, ulceration, and non-flat morphology significantly decreased the diagnostic accuracy of EUS. Furthermore, EGCs that corresponded to the expanded criteria were more likely to be overestimated by EUS.

The detection rate of EGCs has increased with the Korean National Cancer Screening Program. Additionally, the appropriate management plan for ECG is determined according to the depth of tumor invasion. Therefore, accurate T staging is critical for decision-making in EGCs.^3,4^ While MDCT has been conventionally used for TNM staging in gastric cancer, it has low accuracy in T staging, especially in distinguishing mucosal and submucosal tumors. Endoscopic Ultrasonography, which delineates individual gastric wall layers, is considered the most reliable imaging modality.^10^ Furthermore, it has the advantage of placing the transducer close to the lesion without the interference of fat, bowel gas, muscle, or bone.^11^

We found that the accuracy of EUS for overall T staging in EGC was 66.4%, which is comparable to that in a previous systematic review of 22 studies (65%-92.1%).^11^ The accuracy of EUS in this study was lower than what has been observed in other studies, which is attributable to the higher rate of ulceration and larger tumor size. In this study, 36.8% of tumors had ulceration, and 28.3% of lesions were >3 cm, whereas previous studies had average ulceration rates of 7.7%-19.6%, and 17.5%-26.4% of tumors were >3 cm.^16,21-23^ In a study whose rate of lesions larger than 3 cm was identical to that in our study (28.3%), the accuracy of EUS was 41.4%.^12^ Considering the accuracy of EUS relies heavily on ulceration and size, the accuracy of EUS for overall T-staging in our study seems reasonable.

We found that the accuracy of EUS was associated with ulceration, larger size, and non-flat morphology. These have also been reported to be the main factors that decrease the accuracy of EUS in other studies.^12,16,24-26^ This study showed that the presence of ulceration was associated with overestimation by EUS. The rate of ulceration in EGC was reported to be about 10%-30%, and these ulcers cause hypoechoic lesions on EUS due to fibrosis and peritumoral inflammation. Therefore, it is difficult to differentiate whether the ulcer represents tumor invasion or thickening due to fibrosis.^22^ Several studies have suggested that non-flat morphology also reduces the accuracy of EUS. Depressed morphology accompanied by ulcerous change is associated with overestimation, whereas elevated morphology leads to underestimation.^12^ Tsujii et al.^17^ also suggested that an irregular surface, submucosal tumor-like marginal elevation, and fold thickness were associated with poor image quality and incorrect diagnosis. Additionally, tumor size larger than 2 cm leads to the overestimation of pathologic T1a and T1b tumors in that a lesion with a larger size requires more extensive areas to be scanned. Therefore, studies have consistently shown that ulceration, larger size, and non-flat morphology are correlated with a low accuracy of EUS.

However, studies exploring the association of tumor location and histopathologic type with the accuracy of EUS have revealed conflicting results. Tsuzuki et al.^13^ showed that the unfavorable accuracy of EUS in tumors in the upper third of the stomach was due to anatomical characteristics such as the relatively thin submucosal layer, many vessels, and inadequate filling of water. Previous studies also showed that the accuracy of EUS tended to decline for lesions located in the upper third of the stomach.^15,21^ Other studies have shown that the tumor location and the accuracy of EUS were not correlated.^14,22,24,26^ With regard to histopathology, several studies have demonstrated that histopathologic differentiation is not correlated with the accuracy of EUS,^21,24^ whereas other studies have shown a correlation.^16,22,24^ Although undifferentiated lesions decrease the accuracy of EUS by formulating diffuse infiltration and small nests, this study focused solely on EGCs, which do not include tumors that have invaded deeper than the submucosa.^27^ To further investigate the impact of tumor location and histopathologic type on the accuracy of EUS, a large and prospective controlled study is required.

We also examined the accuracy of EUS based on the endoscopic resection criteria. Hirasawa et al.^6^ and Gotoda et al.^7^ clarified the risks of lymph node involvement in EGC based on histopathologic type, size, and depth of invasion and suggested expanded criteria for endoscopic resection. Our results showed that tumors corresponding to the expanded criteria, which include ulceration and larger tumors, were more likely to be overestimated. Therefore, lesions corresponding to the expanded criteria should be carefully considered to determine whether these patients can avoid overtreatment with gastrectomy.

This study has several advantages. First, the study used clinical factors such as ulceration, larger size, and macroscopic type, all of which are most frequently assessed in the clinical setting. Second, the study further divides the factors affecting accuracy into those affecting overestimation and underestimation. Third, the study compares the accuracy of EUS between the absolute and expanded criteria.

However, several factors still pose limitations to our study. First, this study was conducted retrospectively; as a result, we enrolled only patients with EGC who underwent EUS and endoscopic resection or gastrectomy. Second, in some instances, 2 lesions were enrolled from the same patient, which introduces a statistical dependency. However, EUS was performed and analyzed per lesion, not per patient. Additionally, there were only 12 synchronous EGC patients, and therefore the bias is expected to be negligible. Third, the type of EUS probe was not limited or included in the analysis. In this study, a radial probe was mainly used, but a mini-probe could be used without restrictions. The decision on which probe to use was made by the endoscopist.

In conclusion, ulceration, tumor size larger than 2 cm, and non-flat morphology were correlated with decreased accuracy of EUS in EGC patients. This indicates that lesions corresponding to the expanded criteria were more likely to be overstaged by EUS. Therefore, EGCs with ulceration and a size larger than 2 cm should be carefully considered when performing local staging by EUS before gastrectomy.

## Figures and Tables

**Table 1.** Baseline Characteristics of Early Gastric Cancer Patients and Lesions (n = 223)

Characteristics	Number of EGC (%)
**Age, **mean (SD), (years)	62.8 (11.2)
**Gender, n (%)**	
Male	148 (66.4)
Female	75 (33.6)
**BMI **(m^2^/kg)	23.4 (2.8)
**Size of EGC **(cm), **n (%)**	
≦2 cm	101 (45.3)
>2 to ≦3 cm	59 (26.5)
> 3 cm	63 (28.3)
**Location, n (%)**	
Upper third	24 (10.8)
Middle third	82 (36.8)
Lower third	117 (52.5)
**Ulcer, n (%)**	
Present	82 (36.8%)
No	141 (63.2%)
**Initial treatment, n (%)**	
Endoscopic resection (EMR, ESD)	65 (29.1)
Gastrectomy	158 (70.9)
**Histopathologic type, n (%)**	
Differentiated	123 (55.2)
Undifferentiated	100 (44.8)
**Depth of invasion, n (%)**	
Mucosa	134 (60.1)
SM1	30 (13.5)
SM2	59 (26.5)
**Macroscopic types, n (%)**	
Elevated (I + IIa)	22 (9.9)
Flat (IIb)	49 (22.0%)
Depressed (IIc + III)	130 (58.3%)
Mixed (elevated and depressed)	22 (9.9%)

BMI, body mass index; EGC, early gastric cancer; EMR, endoscopic mucosal resection; ESD, endoscopic submucosal dissection.

**Table 2.** Clinical Factors Affecting the Accuracy of EUS for the Depth of Invasion (n = 223)

**Characteristics**	**Accuracy (n = 148)**	**Inaccuracy (n = 75)**	*P*
**Gender, n (%)**			.505
Male	96 (64.9%)	52 (35.1%)	
Female	52 (69.3%)	23 (30.7%)	
**BMI** (m^2^/kg)			.315
<25	45 (71.4%)	18 (28.6%)	
≥25	103 (64.4%)	57 (35.6%)	
**Location, n (%)**			.256
Upper third	19 (79.2%)	5 (20.8%)	
Middle third	56 (68.3%)	26 (31.7%)	
Lower third	73 (62.4%)	44 (37.6%)	
**Ulcer, n (%)**			<.001
Present	41 (50.0%)	41 (50.0%)	
No	107 (75.9%)	34 (24.1%)	
**Endoscopists, n (%)**			.843
A	42 (64.6%)	23 (35.4%)	
B	31 (72.1%)	12 (27.9%)	
C	55 (64.7%)	30 (35.3%)	
D	20 (66.7%)	10 (33.3%)	
**Histopathologic type, n (%)**			.213
Differentiated	86 (69.9%)	37 (30.1%)	
Undifferentiated	62 (62.0%)	38 (38.0%)	
**Depth of invasion, n (%)**			.142
Mucosa	94 (70.1%)	40 (29.9%)	
Submucosa	54 (60.7%)	35 (39.3%)	
**Macroscopic types, n (%)**			<.001
Flat (IIb)	43 (87.8%)	6 (12.2%)	
Non-flat	105 (60.3%)	69 (39.7%)	

EUS, endoscopic ultrasonography; BMI, body mass index.

**Table 3.** Univariate and Multivariate Analysis for Affecting the Accuracy of EUS for the Depth of Invasion

	**Univariate Analysis OR [95% CI]**	*P*	**Multivariate Analysis OR [95% CI]**	*P*
**Size**				
≦2 cm	1		1	
>2 to ≦3cm	3.83 [1.75-8.41]	.001	3.59 [1.65-7.79]	.001
>3 cm	5.74 [2.64-12.48]	<.001	5.47 [2.55-11.74]	<.001
**Location**				
Lower third	1			
Middle third	0.83 [0.42-1.65]	.607		
Upper third	0.35 [0.11-1.12]	.079		
**Ulcer**				
No	1			
Present	2.48 [1.30-4.76]	.006	2.62 [1.38-4.97]	.003
**Histopathologic type**				
Differentiated	1			
Undifferentiated	1.37 [0.72-2.60]	.328		
**Macroscopic types**				
Flat [IIb]	1		1	
Non-flat	2.96 [1.10-7.92]	.030	2.94 [1.11-7.74]	.029

EUS, endoscopic ultrasonography; OR, odds ratio.

**Table 4.** Clinical Factors Affecting the Over- and Underestimation of EUS for the Depth of Invasion

	**Overestimation (n = 59)**	*P*	**Underestimation (n = 16)**	*P*
**Size, n (%)**		<.001		.068^†^
≦2 cm	11/86 (11.5%)		5/90 (5.6%)	
>2 to ≦3 cm	22/55 (40.0%)		4/37 (10.8%)	
>3 cm	26/56 (46.4%)		7/37 (18.9%)	
**Location, n (%)**		.229		.564
Upper third	3/22 (13.6%)		2/21 (9.5%)	
Middle third	22/78 (28.2%)		4/60 (6.7%)	
Lower third	34/107 (31.8%)		10/83 (12.0%)	
**Ulcer, n (%)**		<.001		.242^†^
Present	39/80 (48.8%)		2/43 (4.7%)	
No	20/127 (15.7%)		14/121 (11.6%)	
**Histopathologic type, n (%)**		.107		.735
Differentiated	27/113 (23.9%)		10/96 (10.4%)	
Undifferentiated	32/94 (34.0%)		6/68 (8.8%)	
**Macroscopic types, n (%)**		.001		.239^†^
Flat (IIb)	4/47 (8.5%)		2/45 (4.4%)	
Non-flat	55/160 (34.4%)		14/119 (11.8%)	

^†^Fisher exact test was conducted to test.

EUS, endoscopic ultrasonography.

**Table 5.** Multivariate Analysis for Factors Affecting the Overestimation of EUS for the Depth of Invasion

	**Multivariate analysis OR [95% CI]**	*P*
**Size**		
≦2 cm	1	
>2 to ≦3 cm	4.48 [1.84-10.91]	.001
>3 cm	6.23 [2.58-15.03]	<.001
**Ulcer**		
No	1	
Present	4.37 [2.16-8.94]	<.001
**Macroscopic types**		
Flat (IIb)	1	
Non-flat	2.68 [0.84-8.51]	.094

EUS, endoscopic ultrasonography; OR, odds ratio.
